# Methionine restriction slows down senescence in human diploid fibroblasts

**DOI:** 10.1111/acel.12266

**Published:** 2014-10-01

**Authors:** Rafał Kozieł, Christoph Ruckenstuhl, Eva Albertini, Michael Neuhaus, Christine Netzberger, Maria Bust, Frank Madeo, Rudolf J Wiesner, Pidder Jansen-Dürr

**Affiliations:** 1 Institute for Biomedical Aging Research (IBA), Universität InnsbruckRennweg 10, 6020, Innsbruck, Austria; 2 Institute of Molecular Biosciences, Karl-Franzens UniversityHumboldtstrasse 50, 8010, Graz, Austria; 3 Institute for Vegetative Physiology, University of KölnRobert-Kochstr. 39, 50931, Köln, Germany; 4 Center for Molecular Medicine Cologne, University of KölnRobert-Kochstr. 21, 50931, Köln, Germany; 5 Cologne Excellence Cluster on Cellular Stress Responses in Aging-associated Diseases (CECAD)Joseph-Stelzmannstr. 26, 50931, Köln, Germany

**Keywords:** cellular senescence, fibroblast, methionine, mitochondria, oxidative stress

## Abstract

Methionine restriction (MetR) extends lifespan in animal models including rodents. Using human diploid fibroblasts (HDF), we report here that MetR significantly extends their replicative lifespan, thereby postponing cellular senescence. MetR significantly decreased activity of mitochondrial complex IV and diminished the accumulation of reactive oxygen species. Lifespan extension was accompanied by a significant decrease in the levels of subunits of mitochondrial complex IV, but also complex I, which was due to a decreased translation rate of several mtDNA-encoded subunits. Together, these findings indicate that MetR slows down aging in human cells by modulating mitochondrial protein synthesis and respiratory chain assembly.

## Introduction

Dietary restriction is the only known nongenetic intervention that can extend both lifespan and health span in most if not all species up to mammals (Lopez-Torres & Barja, 2008). Moreover, most of the physiologic, hematologic, hormonal, and biochemical changes produced by dietary restriction in rodents and other animals are also observed in humans and nonhuman primates (Spindler, 2010; Stein *et al*., 2012). Lifespan extension was also observed when the protein content in food was decreased (Pamplona & Barja, 2006), and many of the benefits of protein restriction can be ascribed to the decreased intake of one particular amino acid L-methionine. Accordingly, methionine restriction (MetR) was shown to extend the lifespan of rodents, such as rats and mice, up to 40% (Orentreich *et al*., 1993; Caro *et al*., 2008; Sun *et al*., 2009). It appears that phenotypical responses to MetR are not fully conserved in evolution; for example, a decrease of dietary methionine alone was not sufficient to enhance lifespan in the fruit fly *Drosophila melanogaster*. Instead, methionine restriction was proposed to act in combination with one or more other essential amino acids in flies (Grandison *et al*., 2009).

The mechanisms by which methionine restriction extends lifespan are incompletely understood, and, based on experimental data, two major concepts are emerging: As MetR profoundly decreases reactive oxygen species (ROS) production in rat mitochondria (Sanz *et al*., 2006; Caro *et al*., 2008), it has been hypothesized that lifespan extension by MetR is due to decreased oxidative stress (Lopez-Torres & Barja, 2008). In an alternative concept, it was proposed that MetR extends lifespan by decreasing the protein biosynthesis rate due to methionine limitation (Hipkiss, 2008), consistent with the finding that decreasing the general rate of protein translation can extend lifespan in nematodes (Hansen *et al*., 2007; Syntichaki *et al*., 2007). In yeast, induction of autophagy and stress-responsive retrograde signaling were shown recently to underly lifespan extension by MetR (Johnson & Johnson, 2014; Ruckenstuhl *et al*., 2014).

It is currently unknown if reduced methionine content in food affects the rate of aging in humans and the only available data correspond to genetic modulations of methionine synthesis pathway (Johnson & Johnson, 2014). A convenient way to study basic mechanisms relevant for human aging is to study cellular senescence in cell culture models (Hayflick, 1997; Campisi, 2011). Studies of cellular senescence provided important insights into basic mechanisms of human aging at the cellular level (Bodnar *et al*., 1998; Campisi, 2005) and suggested an important role of mitochondrial dysfunction in this process (Passos *et al*., 2007; Stockl *et al*., 2007). There is increasing evidence that, besides serving as a model to study human aging *in vitro*, cellular senescence plays an important role in organismic aging *in vivo* (Baker *et al*., 2011; Campisi, 2011). In the present work, we addressed the question whether reduced methionine availability in the culture media can postpone replicative senescence of human diploid fibroblasts (HDF), and if so, whether alterations of mitochondrial function would contribute to this process.

## Results

### MetR slows down senescence of HDF

**Figure 1:**
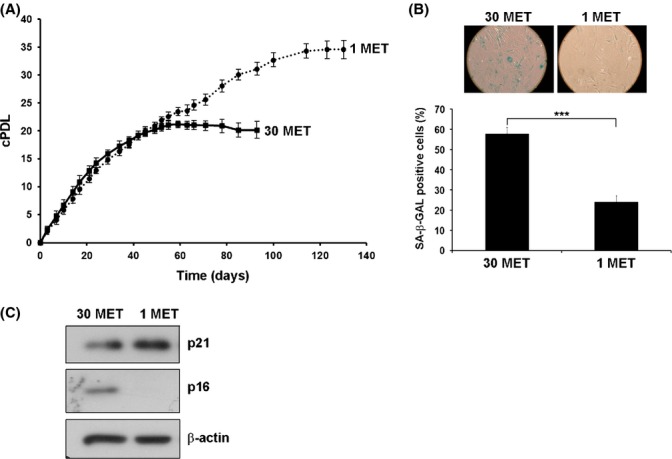
Methionine restriction extends the replicative lifespan of human diploid fibroblasts. (A). Human diploid fibroblasts were grown in DMEM containing either 30 mg L^−1^ (30 MET) or 1 mg L^−1^ (1 MET) of methionine, as indicated. The number of cumulative population doublings (cPDL) was calculated at regular intervals, as described in Material and methods. Growth curves representing three independent experiments are shown. (B). Cells treated as in panel A were stained at day 50 for SA-ß-gal activity (top panel). Lower panel represents results (mean ± SE) obtained from three independent experiments. (C). Lysates were prepared from cells treated as in panel A at day 50, separated by SDS-PAGE, and stained with antibodies to p21^Cip1/Waf1^ and p16^INK4A^, as indicated. ß-actin was used as loading control. One of three representative blots is shown. *** P < 0.001.

To assess the influence of methionine restriction on the proliferative lifespan of HDF, cells were cultured under standard conditions (i.e. in the presence of 30 mg L^−1^ methionine), or grown in media with decreased methionine concentration. Stepwise lowering of the methionine concentration, down to 1 mg L^−1^, had no significant effect on the rate of cell proliferation in early passage cells (Table1). Complete omission of methionine from the cell culture medium significantly impaired cell proliferation (Table1), indicating that a certain level of exogenous methionine is required for full proliferative capacity of such cells. To avoid confounding effects of methionine shortage on cell proliferation, we chose a methionine concentration of 1 mg L^−1^ for MetR experiments. With continued passaging, the rate of cell proliferation gradually decreased around day 60 in cultures grown in standard medium (containing 30 mg L^−1^ methionine), whereas cells cultivated under MetR were still actively proliferating until day 120; from the onset of treatment, control cells completed 21 cumulative population doublings (cPDL), whereas MetR cells completed 34 cPDL (Fig.1A). Cells grown in the presence of 30 mg L^−1^ methionine displayed a marked increase in the percentage of cells positive for the senescence-associated ß-galactosidase (SA-ß-gal) relative to MetR cells (Fig.1B). Increased senescence in these cultures was confirmed by increased expression of the senescence marker p16^INK4a^, whereas expression of p21^Cip1/Waf1^, an indicator of cell cycle progression, was not significantly affected by methionine restriction (Fig.1C). Together, these data indicate that MetR induces a significant extension of the replicative lifespan of HDF, increasing the number of cumulative population doublings (cPDL) by roughly 40%. It should be noted that all functional experiments were performed at the same day with control vs. MetR cells, irrespective of the elapsed cPDL, to allow direct comparison of both cell types in the same assay.

**Table 1:** Effect of decreased methionine concentration on proliferation of HDF

Methionine concentration (mg L^−1^)	0	1	6	30
cPDL at day 20	3.3 ± 0.5	11.4 ± 0.2	13 ± 0.1	12.9 ± 0.2
cPDL at day 50	7.8 ± 0.3	20.9 ± 0.6	20.7 ± 0.3	20.2 ± 0.4
cPDL at day 70	11.6 ± 0.5	25.6 ± 0.7	23.1 ± 0.5	21.0 ± 0.4

Human diploid fibroblasts were grown in DMEM containing either 30 mg L^−1^ (regular DMEM), 6 mg L^−1^, 1 mg L^−1^, or 0 mg L^−1^ of methionine, as indicated. Cells were counted in regular intervals, and cumulative population doublings were calculated. Results (mean ± SE) of three independent experiments are shown.

### Decreased ROS levels in MetR cells

**Figure 2:**
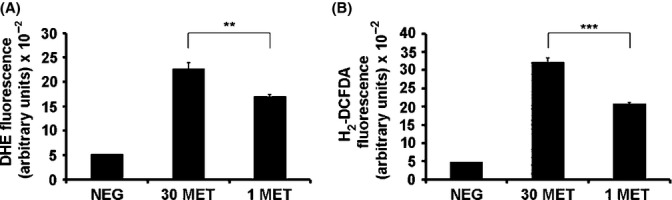
Methionine restriction decreases ROS levels in HDF. Human diploid fibroblasts were grown in DMEM containing 30 mg L^−1^ or 1 mg L^−1^ of methionine, as indicated. At day 30, cells were not stained (NEG) or stained with DHE (A) or H_2_DCFDA (B) as indicated, and fluorescence intensity was determined by flow cytometry. ** P < 0.01, *** P < 0.001.

To assess changes in cellular ROS production, cells were stained with redox-sensitive fluorescent dyes and relative fluorescence was quantitated. A variety of such probes are available; however, none of them provide true quantitative data of ROS levels, and for technical reasons, it is not entirely clear which probe detects which radical species; accordingly, several probes should be used to get an estimate of intracellular ROS levels (Forkink *et al*., 2010). As general markers of ROS, cells were stained with dihydroethidium (DHE) and 2'-7'-dichlorodihydrofluorescein diacetate (H_2_DCFDA), respectively. In both cases, fluorescence was significantly decreased in MetR cells (Fig.2), indicating that ROS levels are lower in MetR fibroblasts.

### MetR decreases mitochondrial activity

**Figure 3:**
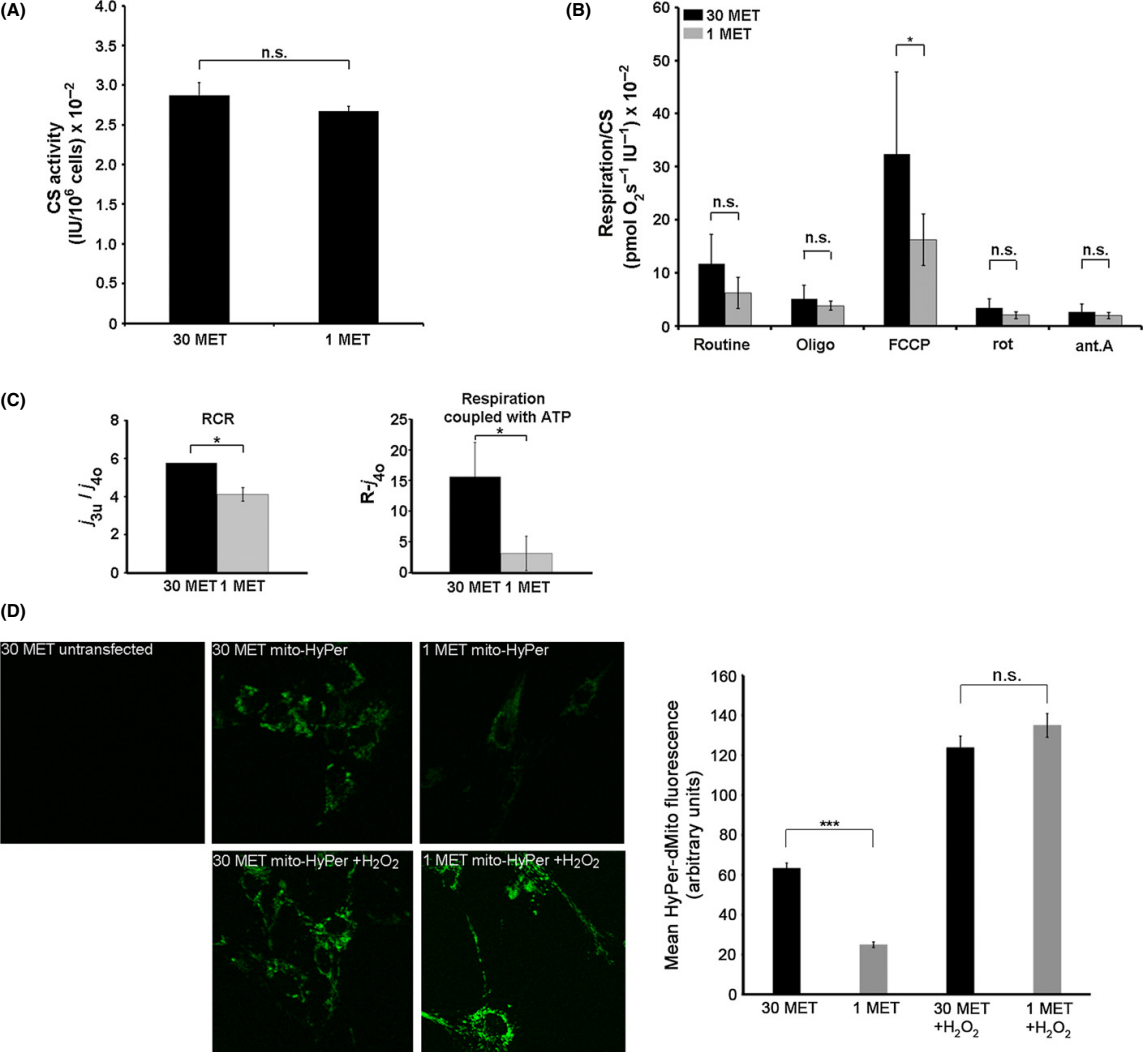
Methionine restriction leads to downregulation of mitochondrial respiratory activity. (A). Human diploid fibroblasts were grown in DMEM containing 30 mg L^−1^ or 1 mg L^−1^ of methionine as indicated. Cells were harvested after 35 days, and citrate synthase activity was determined in cellular lysates (mean ± SE, *n* = 4). (B). Cells were treated as in panel A harvested after 35 days, and oxygen consumption was determined immediately in an Oxygraph 2k for untreated cells (routine) or cells after the subsequent addition of oligomycin (oligo), FCCP, rotenone (rot) and antimycin A (ant.A) as indicated (mean ± SE, *n* = 4). (C). Data shown in panel B were used to calculate for each culture condition the respiratory control ratio (RCR) and the respiratory activity coupled to ATP production. The respiratory control ratio (RCR) was calculated as a ratio of uncoupled respiration (after addition of FCCP, *j*_3u_) to oligomycin-inhibited respiration (*j*_4o_); the rate of respiration coupled with ATP production was determined as a difference between routine (R) and oligomycin-inhibited respiration (*j*_4o_). (D). Human diploid fibroblasts were infected with lentiviral particles harboring a HyPer-dMito expression vector. Cells were grown in DMEM containing 30 mg L^−1^ or 1 mg L^−1^ of methionine, as indicated. 30 days after infection, HyPer-dMito fluorescence was analyzed using confocal microscopy. Uninfected cells were used as negative control, and cells treated for 1 h with 100 μm H_2_O_2_ were used as positive control. One representative experiment is shown (left panel); right panel shows densitometry data obtained from three independent experiments. * P < 0.05, *** P < 0.001.

Next, we addressed the question whether altered mitochondrial function contributes to cellular lifespan extension by MetR. We found that decreasing the methionine concentration did not affect the activity of citrate synthase (Fig.3A), a widely used marker for mitochondrial mass (Holloszy *et al*., 1970; Perrone *et al*., 2010). The rate of maximal respiration after uncoupling by FCCP normalized to mitochondrial mass was significantly decreased in MetR fibroblasts (Fig.3B), indicating a decreased maximal activity of the mitochondrial respiratory chain. This was accompanied by a decreased respiratory control ratio (RCR, referred to as *j*_3u/4o_), measured in intact cells as the ratio of uncoupled (state 3u, FCCP treated) to the oligomycin-inhibited (state 4o) flux (Fig.3C). The RCR is strongly influenced by almost every functional aspect of oxidative phosphorylation, making them a good indicator of mitochondrial dysfunction (Brand & Nicholls, 2011). The observed decrease in RCR indicates partial uncoupling in MetR cells, which may lead to a diminished oxidative ATP production. Subtraction of oligomycin-inhibited respiration from routine respiration (R-*j*_*4o*_) allowed to determine the part of mitochondrial respiratory activity coupled with ATP production. We observed a significant decrease in respiration coupled with ATP synthesis in MetR cells (Fig.3C). Accordingly, decreased activity of the mitochondrial respiratory chain was accompanied by a decreased level of mitochondrial H_2_O_2_ (Fig.3D), visualized by HyPer-dMito, a mitochondria targeted sensor protein specific for H_2_O_2_ (Belousov *et al*., 2006), and quantitated by densitometric analysis.

**Figure 4:**
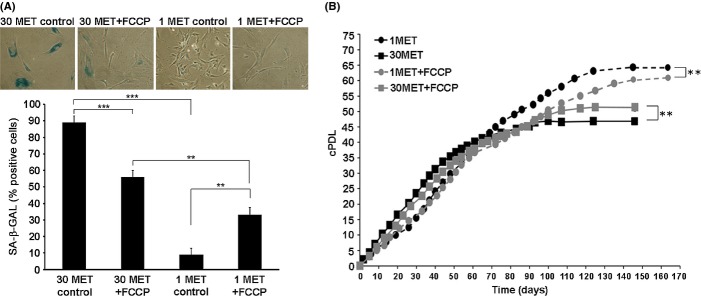
Mitochondrial uncoupling decreases lifespan extension by MetR. (A). Human diploid fibroblasts were grown in DMEM containing 30 mg L^−1^ or 1 mg L^−1^ of methionine, as indicated. Where indicated, the medium was additionally supplemented with 2 μmFCCP. After 90 days, the cells were stained for SA-ß-gal activity. Results (mean ± SE) of three independent experiments are shown. (B). Cells were prepared as in panel A, cultivated for 170 days, and growth curves were calculated out of three independent experiments (mean ± SE). ** P < 0.01, *** P < 0.001.

Together, these findings suggest that mitochondria are partially uncoupled in MetR cells and raise the possibility that extended lifespan of MetR cells is due to mild mitochondrial uncoupling, consistent with earlier findings that mild uncoupling of mitochondria has the potential to extend lifespan of human fibroblasts (Passos *et al*., 2007). In agreement with this assumption, chronic exposure of cells to low doses of the chemical uncoupler carbonyl cyanide 4-(trifluoromethoxy) phenylhydrazone (FCCP) delayed the onset of senescence in control cells (Fig.4). However, exposure to FCCP significantly reduced the positive effects of MetR on both the proliferative capacity (Fig.4B) and entry into senescence (Fig.4A) of HDF. Apparently, further uncoupling of mitochondria decreased lifespan in MetR cells, consistent with earlier findings that chronic exposure of HDF to higher concentrations of FCCP decreases their proliferative capacity, probably due to insufficient ATP production (Stockl *et al*., 2007).

### MetR inhibits OxPhos complex IV

**Figure 5:**
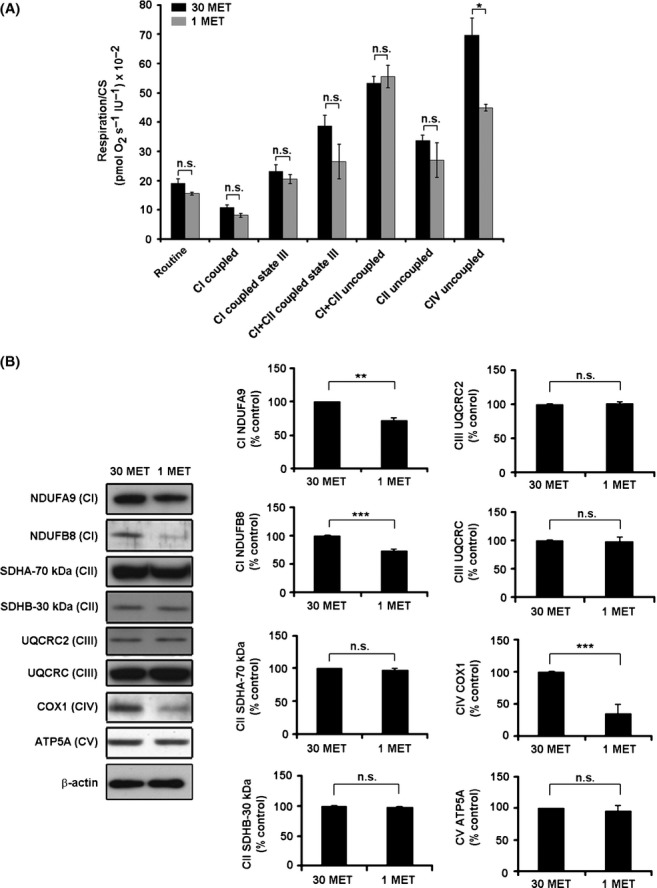
Decreased level and activity of respiratory chain complex IV in methionine-restricted cells. A. Human diploid fibroblasts were grown in cell culture medium containing 30 mg L^−1^ or 1 mg L^−1^ of methionine as indicated. Cells were harvested after 33 days, and oxygen consumption was determined using Oxygraph 2k using untreated cells (routine) or cells permeabilized after addition of digitonin. The rate of respiration was determined after addition of malate and glutamate (complex I coupled respiration), ADP (complex I state III respiration), succinate (complex I + complex II state III respiration), FCCP (complex I + complex II uncoupled respiration), rotenone (complex II uncoupled respiration) and finally antimycin A, ascorbate and TMPD (CIV uncoupled respiration). The data were normalized to mitochondrial mass marker citrate synthase activity. Results (mean ± SE) obtained from four independent experiments are shown. B. Cells were treated as in panel A, lysed at day 33, and the expression of selected subunits of OxPhos complexes I through V was analyzed by Western blot, as indicated. Left panel represents one typical experiment; right panel shows the results (mean ± SE) obtained from three independent experiments. ß-actin signal was used for normalization. ** P < 0.01, *** P < 0.001.

When respiration was measured in permeabilized cells using different substrates and inhibitors of the respiratory chain complexes, we observed a significant decrease in the respiratory activity of mitochondrial complex IV in MetR cells, whereas the activity of the other oxidative phosphorylation system (OxPhos) complexes was not significantly altered (Fig.5A). Furthermore, the abundance of the index subunit COX1 of complex IV as well as two index subunits (NDUFA9 and NDUFB8) of complex I was decreased in MetR cells (Fig.5B), whereas subunits of other complexes were unchanged, suggesting that altered stoichiometry of OxPhos complexes may contribute to the altered respiratory profile of MetR cells.

### Differential translation rate of OxPhos subunits in MetR cells

**Figure 6:**
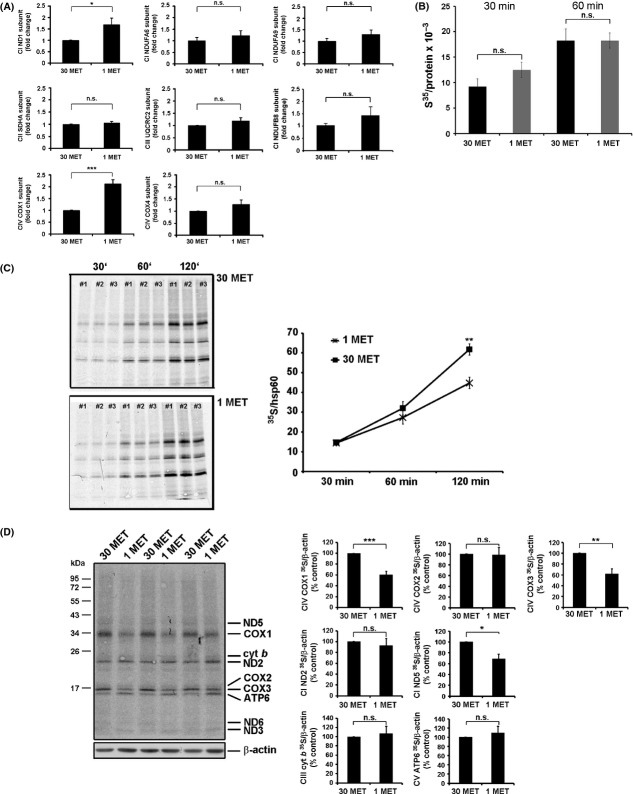
Post-transcriptional regulation of OxPhos complexes in MetR cells. (A). Human diploid fibroblasts were grown in DMEM containing 30 mg L^−1^ or 1 mg L^−1^ of methionine as indicated. mRNA levels for selected subunits of OxPhos complexes were determined by qPCR after 33 days of treatment, as indicated (mean ± SE, *n* = 3). (B). Cells were treated as in panel A, and total cellular protein synthesis was measured by ^35^S-Methionine incorporation for 30 min and 60 min. The data are presented as means ± SE (C). Cells were treated as in panel A, and total mitochondrial protein synthesis was measured in the presence of emetine by ^35^S-Methionine incorporation for 30 min, 60 min, and 120 min. Left panel shows original data; right panel shows results (mean ± SE) obtained from three independent experiments. (D). Cells were treated as in panel A, and the rate of synthesis of individual mitochondrial subunits was analyzed by ^35^S-Methionine incorporation for 60 min. Incorporation rates were normalized to ß-actin used as a loading control. Left panel shows original data; right panel shows results (mean ± SE) obtained from three independent experiments. * P < 0.05, ** P < 0.01, *** P < 0.001.

mRNA levels for several nuclear-encoded mitochondrial subunits of OxPhos complexes, such as NDUFA6, NDUFA9, and NDUFB8 (complex I), SDHA (complex II), UQCRC2 (complex III), or COX4 (complex IV) were not altered when comparing control and MetR HDF (Fig.6A). mRNA levels for COX1 and ND1, two mitochondrially encoded subunits, were even increased in MetR cells. Together, these findings indicate that the decreased protein levels of complex I and IV subunits in MetR cells are due to post-transcriptional regulation. When protein synthesis was measured by ^35^S-Methionine incorporation into total cellular protein, no significant differences were found (Fig.6B). However, as shown in the presence of the cytosolic ribosome inhibitor emetine, the incorporation of ^35^S-Methionine into mitochondrial DNA-encoded subunits was significantly decreased in MetR cells (Fig.6C). The rate of synthesis for OxPhos subunits COX1, COX3, and ND5 was significantly decreased in MetR cells, while it was slightly increased for mitochondrially encoded OxPhos subunits cytochrome *b* and ATP synthase 6 (Fig.6D). Other subunits were not ^35^S-labeled sufficiently to allow quantification.

## Discussion

Methionine restriction (MetR) is a well-established and robust protocol for lifespan extension in rodents (Sanz *et al*., 2006) and was shown to significantly improve metabolic health in these animals. Thus, MetR decreased oxidative damage in rat brain (Naudi *et al*., 2007), decreased visceral fat mass and preserved insulin action in aging rats (Malloy *et al*., 2006), increased mitochondrial aerobic capacity in liver and skeletal muscle (Perrone *et al*., 2010), and enhanced metabolic flexibility in both fed and fasted rats (Hasek *et al*., 2010). Similarly, it was recently shown that MetR increased fat oxidation in obese humans with metabolic syndrome (Plaisance *et al*., 2011); however, the effects of methionine restriction in food on aging in humans remained elusive and the only available data correspond to genetic manipulations of methionine synthesis pathway (Johnson & Johnson, 2014). Recently published data in unicellular fungi indicate induction of autophagy as an alternative mechanism for lifespan extension by MetR (Ruckenstuhl *et al*., 2014). However, we do not think that autophagy induction contributes to lifespan extension observed by MetR in human diploid fibroblasts, as MetR was still able to extend lifespan in HDF depleted for Beclin-1, an essential mediator of autophagy in human cells (Fig. S1). Whereas our finding that MetR reduces intracellular ROS levels in HDF suggests that lifespan extension by MetR may at least in part due to reduced oxidative stress, as was suggested for MetR in rodents (Naudi *et al*., 2007), the causality of decreased ROS for lifespan extension in HDF is not formally established by our experiments.

Here, we report for the first time that low methionine content can postpone senescence in a human cellular model. We found that in MetR cells, the activity of oxidative phosphorylation (OxPhos) complex IV is specifically decreased, due to downregulation of translation of complex IV subunits encoded by mitochondrial DNA. This led to lower steady state levels of the complex IV subunit COX1, but also of subunits for complex I, which are all assembled *in situ* into higher order supercomplexes stabilizing each other (Schagger, 2002; Hornig-Do *et al*., 2012). As COX1 and ND1 mRNA levels, both encoded in mitochondrial DNA, were even increased and mRNA levels for nuclear-encoded subunits were unchanged, our data suggest a complex translational control of subunit synthesis and assembly in MetR cells. The most easy explanation, namely a general slowing of mitochondrial protein synthesis under MetR, could not be confirmed, as the rate of synthesis of cytochrome *b* and ATP synthase 6, both encoded in mitochondrial DNA, was not altered in MetR cells. We postulate that under MetR, the mitochondrial Met pool decreases, to preferentially satisfy the cytosolic ribosomes. It is well accepted that most cells can live with less OXPHOS, in cell culture but also in *vivo* (Wredenberg *et al*., 2002; Dufour *et al*., 2008), while impaired cytosolic protein synthesis can be lethal (Ch'ih *et al*., 1976).

Data reported here clearly indicate that MetR decreased the respiratory control ratio and the rate of respiration coupled to ATP production in human diploid fibroblasts, suggesting that MetR induces mild mitochondrial uncoupling. This observation may explain the positive effects of MetR on lifespan of HDF, as pharmacological uncoupling of the mitochondria by dinitrophenol was shown to postpone senescence and extend lifespan in this cell type as well (Passos *et al*., 2007). In agreement with this conclusion, MetR cells contained significantly decreased mitochondrial H_2_O_2_ levels, representing a well-known consequence of mitochondrial uncoupling (Mailloux & Harper, 2012). It is known that changes in the architecture and function of OxPhos complexes lower mitochondrial membrane potential and ATP production in genetic mitochondrial diseases affecting complex IV subunits (Szczepanowska *et al*., 2012), and small hairpin RNA-mediated knockdown of complex IV subunits induced mitochondrial uncoupling in human cells (Galati *et al*., 2009; Fornuskova *et al*., 2010). Accordingly, we hypothesize that mild mitochondrial uncoupling observed in HDF under MetR results from MetR-induced changes in the stoichiometry and activity of OxPhos complexes IV and I. As reduced activity of complex IV appears central to the lifespan extension by MetR, we attempted to specifically reduce COX1 levels, a complex IV subunit encoded by the mtDNA. There is no published procedure to knock down the expression of proteins encoded in mitochondrial DNA, and classical shRNAs systems are not operative in the mitochondria due to the lack of the Dicer-processing machinery. Hence, we followed the idea that a reduction of COX4, a nuclear-encoded ETC subunit forming an essential part of the complex IV assembly line, may in consequence lead to a depletion of other subunits (including mtDNA-encoded COX1) of the same complex, as unassembled subunits are rapidly degraded (Hornig-Do *et al*., 2012). Using this strategy, we observed a coordinated reduction in the concentration of both complex IV subunits, the silenced nuclear-encoded subunit COX4 as well as the mtDNA-encoded subunit COX1, a few days after transfection (data not shown), indicating that this approach is in principle feasible. However, the knockdown of subunit 4 was not stable, and levels recovered after about two weeks; we also observed a compensatory response whereby COX1 levels were upregulated after two weeks, even exceeding the COX1 levels in the control cells. As expected, transient and short-term COX1 deficiency was not sufficient to alter the lifespan of HDF cells under these conditions (data not shown).

Several studies have addressed mechanisms of lifespan extension by MetR in rodents, mostly investigating gross biochemical alterations induced by MetR in several tissues. Of interest, lifespan extension by MetR has been associated with increased expression of mitochondrial uncoupling protein 1 (UCP1) in the brown adipose tissue of both rats (Hasek *et al*., 2010) and mice (Plaisance *et al*., 2010). This finding suggests that beneficial effects of MetR in rodents may be related at least in part to UCP1 expression in brown fat. However, this cannot be the sole mechanism by which MetR affects aging, as expression of UCP1 was not detectable in HDF under control or MetR conditions (data not shown). Similar to findings reported by Passos *et al*. (2007), we observed, using qRT–PCR, a substantial upregulation of UCP2 mRNA expression in senescent HDF under regular cell culture conditions, that is in media containing 30 mg mL^−1^ of methionine. However, upregulation of UCP2 mRNA was strongly inhibited in HDF under MetR (data not shown), suggesting that mitochondrial uncoupling in MetR cells is most likely independent of UCP2. The reliability of commercially available antibodies to UCP2 is controversial, and we were unable to detect any UCP2 protein in HDF lysates by Western blot, using commercially available anti-UCP2 antibodies recommended by others (Mailloux *et al*., 2012). Moreover, whether UCP2 acts as a bona fide uncoupling protein is a matter of intensive debate in the field (Mailloux & Harper, 2012). Taken together, the available data argue against a contribution of both UCP1 and UCP2 to lifespan extension by MetR.

Whereas distinct changes in the abundance of several OxPhos complexes were observed in rat liver under MetR (Sanz *et al*., 2006; Naudi *et al*., 2007), they were not consistent with changes observed in other tissues of the same animals (Caro *et al*., 2009). As it is currently not clear which tissue(s) contributes to the lifespan effects of MetR in rodents, these findings are difficult to interpret at the molecular level. Moreover, one has to take into account that each of the studied tissues is composed of a variety of different cell types, which may differ considerably in their metabolic profile.

The work described here provides a model for cell autonomous effects of MetR and thereby complements *in vivo* studies in rodents. It is a particular strength of the cell-based MetR model used here that changes in replicative lifespan in response to MetR can be directly associated with a single type of mitochondrial alteration, that is UCP-1 independent mild uncoupling of the ETC. Using this model, we propose changes in the control of mitochondrial protein synthesis as key factor responsible for delayed cellular aging under MetR, although additional research is warranted to further address this topic. Our finding that the activity of complex IV decreased in MetR cells is in striking contrast to findings by others who demonstrated that lifespan extension in flies under protein restriction was associated with enhanced mitochondrial activity, increased activity of OxPhos complexes I and IV, and an increased translation rate for several complex I and IV subunits (Zid *et al*., 2009). Together, these observations suggest that different protocols of dietary restriction can extend lifespan through opposite effects on mitochondrial function, consistent with the hypothesis that an optimal level of mitochondrial OxPhos activity can be defined for maximal lifespan of a given model system, and, from there, any increase or decrease of mitochondrial activity will lead to lifespan shortening (Rea *et al*., 2007).

Taken together, the results reported here demonstrate that reduced methionine content in culture media extends the replicative lifespan of human fibroblasts and suggest that specific alterations in mitochondrial protein synthesis and respiratory chain assembly contribute to lifespan extension. These findings extend our mechanistic understanding of how MetR can influence aging at the cellular level. It will be interesting to determine whether changes in mitochondrial protein synthesis contribute to lifespan extension by MetR in animal models.

## Experimental procedures

### Chemicals

All chemicals were purchased from Sigma-Aldrich (Vienna, Austria), unless indicated otherwise.

### Cell culture

Human foreskin fibroblasts (HFF-2) pooled from four newborn were purchased from the American Type Culture Collection (Manassas, VA, USA) at a passage number of 6 (roughly 14–16 cPDL) and were used for the experiments described here starting from roughly 20 cPDL. Cells were maintained in Dulbecco's modified Eagle's medium (DMEM, cat. # D0422, Sigma-Aldrich, Vienna, Austria) as described (Hutter *et al*., 2004). The cells were subcultured in an atmosphere of 5% CO_2_ in air at 37 ^°^C using Thermo Scientific HERA cell 150 incubator by passaging them at a ratio of 1:5 at regular intervals, when cells reached 80–90% of confluency. For passaging aged cells, the splitting ratio was progressively decreased to 1:3 and 1:2.

L-cysteine (62.6 mg L^−1^) and L-methionine were added separately to obtain different L-methionine concentrations (30, 6, 1, and 0 mg L^−1^ L-methionine). Cells were counted by a CASY cell counter (Schärfe Systems, Reutlingen, Germany) when passaged, and cumulative population doublings (cPDL) were calculated using the following equation: cPDL = ]Blog(A) – log(B)]/0.301 (A: number of cells at the end of one passage; B: number of cells that were seeded at the beginning of one passage).

### Pulse experiments

Cells were labelled with ^35^S-methionine (0.2 mCi/plate, Hartmann Analytic, Braunschweig, Germany) for 30, 60, and 120 min in methionine- and cysteine-free DMEM supplemented with 100 μg mL^−1^ of the cytoplasmic translation inhibitor emetine and 5% dialyzed fetal bovine serum (Chomyn, 1996). Emetine was omitted to estimate total cellular protein synthesis rate. 25 μg of cell protein were electrophoresed through a 15% denaturing gel. Band intensities corresponding to mitochondrial translation products were quantified densitometrically, using the 30 min time point to ensure that the rate of incorporation was in the linear range. In these experiments, proteins were identified by their molecular mass after autoradiography, which allows unambiguous identification of all mitochondrially encoded proteins (Hornig-Do *et al*., 2012).

### Staining for senescence-associated ß-galactosidase (SA-ß-gal)

The senescent status of the cells was monitored by *in situ* staining for SA-ß-gal, as described (Unterluggauer *et al*., 2003).

### PCR-based quantification of mRNA levels

mRNA levels were determined by qRT–PCR as described before (Lener *et al*., 2009). Absolute quantification of mitochondrial respiratory chain complexes expression was based on a dilution range of an external plasmid standard to obtain a standard curve cycle threshold (Ct) values of the standard versus gene copy numbers. Ct values for mitochondrial respiratory chain complexes in HDF were extrapolated against this plot to calculate absolute copy numbers of mitochondrial respiratory chain complexes mRNA.

### Standard immunoblotting analysis

Cellular protein lysates were prepared on ice in RIPA buffer containing protease and phosphatase inhibitors. Lysates were centrifuged, and protein concentration in the resulting supernatants was determined, using the ‘BCA Protein Assay' (Pierce, Rockford, IL, USA). Appropriate amounts of protein were subjected to SDS gel electrophoresis (8% or 10% SDS/polyacrylamide gels) and transferred to polyvinylidene difluoride (PVDF) membranes. Membranes were blocked with 5% bovine serum albumin in Tris-buffered saline containing 0.1% Tween 20 and incubated with primary antibodies overnight at 4 °C. Proteins of interest were detected after incubation with horseradish peroxidase-conjugated secondary antibodies (Dako Cytomation, Glostrup, Denmark) and visualized with enhanced chemoluminiscence reagent ECL (GE Healthcare, Buckinghamshire, UK). Used antibodies were as follows: anti-complex I NDUFA9 subunit (Invitrogen, Camarillo, CA, USA), anti-complex II 70 kDa subunit (Invitrogen), anti-complex III UQCRC1 subunit (Invitrogen, Frederick, MD, USA), anti-complex IV COX1 subunit (Invitrogen), anti-complex IV subunit IV (Invitrogen), anti-complex V subunit α (Invitrogen), Total OXPHOS Rodent WB Antibody Cocktail (Abcam, Cambridge, UK), anti-p21^Cip1/Waf1^ (BD Pharmingen, San Jose, CA, USA), anti-p16^INK4A^ (Pharmingen, San Jose, CA, USA), anti-β-actin (Sigma, St.Louis, MO, USA). Results from three independent experiments were analyzed by Western blot, and the intensity of the bands was quantitated by densitometry, using AlphaInnotech FluorChem® HD2 instrument (Alpha Innotech, San Leandro, CA, USA) and analyzed using alphaeasefc software, provided by the manufacturer.

### High-resolution respirometry

The respirometry of intact as well as permeabilized cells was performed using an Oxygraph 2K instrument (OROBOROS GmbH, Innsbruck, Austria), as described before (Koziel *et al*., 2013). The respirometry data were normalized to the mitochondrial mass marker enzyme citrate synthase (CS) activity (Kuznetsov *et al*., 2002).

### Assessment of mitochondrial hydrogen peroxide

Production of lentiviral particles was carried out according to the manufacturer's protocol (Addgene Inc., Cambridge, USA) by usage of the packaging plasmids pMD2.G and psPAX2 (Invitrogen) and the lentiviral vector pLenti6/V5-DEST Gateway vector (Invitrogen, Paisley, UK), containing pHyPer-dMito (Evrogen, Moscow, Russia) and control expression sequence, respectively. For lentiviral infection, HDF were cultivated in 6-well plates. Upon reaching ∼70% confluence, culture medium, containing lentiviral particles to the amount of 2 MOI, was added to the cells in presence of 8 μg mL^−1^ hexadimethrine bromide, which increases the efficiency of viral infection. Twenty-four hours after infection, medium was changed. Blasticidin selection was initiated (10 μg mL^−1^) 48 h postinfection. For the detection of mitochondrial H_2_O_2_ levels, the cells were transfected with control or pHyPer-dMito/pLenti6/V5-DEST Gateway lentiviral vector and after expansion analyzed using confocal microscopy or flow cytometry (FACS Canto II, Becton Dickinson, Franklin Lakes, USA). The level of mitochondrial H_2_O_2_ was estimated as a mean value of green pHyPer-dMito fluorescence in 10^4^ cells.

### Primers used for qPCR

For quantitative real-time PCR (qRT–PCR), primers for the detection of mitochondrial respiratory chain complexes mRNA and the housekeeper gene beta-2-microglobulin (B2M) and glyceraldehyde 3-phosphate dehydrogenase (GAPDH) were designed using primer3 software (www.simgene.com), as follows: 5′-CCTACTAACCAACACACTAACC-3′ (fwd) and 5′-CCTGCGAAGAAAAAAACTTCTG-3′ (rev) for NADH dehydrogenase (ubiquinone) 1 alpha subcomplex 9 (NDUFA9), 5′-CAGGGTGGTTGATCTTCTGG-3′ (fwd) and 5′-TAACATGTGTCCGCTGCTTC-3′ (rev) for NADH dehydrogenase 1 alpha subcomplex (NDUFA6), 5′-TGAACTGGGGTGAACCGATG-3′ (fwd) and 5′-AAAGCCAGGAAACCGAAGAG-3′ (rev) for NADH dehydrogenase (ubiquinone) 1 beta subcomplex 8 (NDUFB8), 5′-AGAACATCGGAACTGCGACT-3′ (fwd) and 5′-TTTTCCCACAACCTTCTTGC-3′ (rev) for succinate dehydrogenase complex, subunit A (SDHA), 5′-TTCTTACATGCCACCATCCA-3′ (fwd) and 5′-CTTGCTGCCATTGACTTCTG-3′ (rev) for ubiquinol-cytochrome *c* reductase core protein II (UQCRC2), 5′-GCTCGTTATCATGTGGCAGA-3′ (fwd) and 5′-TCATGTCCAGCATCCTCTTG-3′ (rev) for cytochrome *c* oxidase subunit IV isoform 1 (COX4I1), 5′-ACTACAACCCTTCGCTGACG-3′ (fwd) and 5′-GCGGTGATGTAGAGGGTGAT-3′ (rev) for NADH dehydrogenase subunit 1 (ND1), 5′-ACGTTGTAGCCCACTTCCAC-3′ (fwd) and 5′-TGGCGTAGGTTTGGTCTAGG-3′ (rev) for cytochrome *c* oxidase subunit I (COX1), 5′-GAATTCACCCCCACTGAAAA-3′ (fwd) and 5′-CTCCATGATGCTGCTTACA-3′ (rev) for B2M 5′-GAGTCAACGGATTTGGTCGT-3′ (fwd) and 5′-GATCTCGCTCCTGGAAGATG-3′ (rev) for GAPDH. qPCR was performed on a LightCycler 480 II (Roche, Indianapolis, USA). Cycling conditions were as follows: 95 °C for 8 min (initial denaturation step) followed by 55 cycles of target amplification (95 °C for 15 s, 57 °C for 8 s, and 72 °C for 15 s) and final melting (95 °C for 1 min, 60 °C for 30 s, 95 °C continuous with five acquisitions per °C). Crossing Points (Ct) for mitochondrial complexes and B2M or GAPDH in control cells/methionine-restricted cells were used for calculation of mitochondrial respiratory chain complexes fold expression changes (Unterluggauer *et al*., 2003).

### Assessment of reactive oxygen species (ROS)

The level of the intracellular ROS was measured *in situ* using flow cytometry in cells stained with the dihydroethidium (DHE) or 5-(and-6)-chloromethyl-2',7'-dichlorodihydrofluorescein diacetate (H_2_DCFDA) fluorescent probes (Invitrogen, Oregon, USA). The cells (2 × 10^5^) were trypsinized and rinsed in prewarmed DMEM (for DHE) or HBSS (for H_2_DCFDA), before loading with 20 μm DHE or 10 μm H_2_DCFDA for 30 min at 37 °C. After washing, cells were resuspended in 500 μL PBS and analyzed by flow cytometry on a FACS Canto II (Becton Dickinson, Franklin Lakes, USA). The level of ROS was estimated as a mean value of DHE or H_2_DCFDA fluorescence in 10^4^ cells.

### Citrate synthase activity measurement

Two portions of 300 μL of the sample were taken from the cell suspension stirred in the oxygraph chamber before the chamber was closed for recording respiration. Samples were frozen in liquid nitrogen and stored at −80 °C. Total cell lysate (100 μL) was added to 900 μL of medium containing 0.1 mm 5,5-dithio-bis-(2-nitrobenzoic) acid, 0.5 mm oxaloacetate, 50 μm EDTA, 0.31 mm acetyl-CoA, 5 mm triethanolamine hydrochloride, and 0.1 m Tris/HCl (pH 8.1). The activity of CS was measured spectrophotometrically at 412 nm and 30 °C (Cossarizza *et al*., 1993).

### Statistics

All analyses were performed with the datlab/bd facsdiva software (BD Biosciences, San Jose, CA, USA). All experiments were performed in three independent biological replicates, the results of which were averaged. Differences in cellular characteristics between the group of 30 mg L^−1^ methionine (control) and methionine-restricted cells (*n* = three each) were compared by Student's *t*-test and were represented as mean ± SE. n.s., not significant; * *P* < 0.05; ** *P* < 0.01; *** *P* < 0.001.

## Abbreviations

B2M: beta-2-microglobulin
COX1: cytochrome c oxidase subunit I
COX3: cytochrome c oxidase subunit III
COX4: cytochrome c oxidase subunit IV
CS: citrate synthase
cPDL: cumulative population doublings
DHE: dihydroethidium
DMEM: Dulbecco's modified Eagle's medium
FCCP: carbonyl cyanide 4-(trifluoromethoxy) phenylhydrazone
GAPDH: glyceraldehyde 3-phosphate dehydrogenase
H_2_DCFDA: 5-(and-6)-chloromethyl-2',7'-dichlorodihydrofluorescein diacetate
HDF: human diploid fibroblasts
HFF-2: human foreskin fibroblasts
MetR: methionine restriction
ND1: NADH dehydrogenase, subunit 1
ND5: NADH dehydrogenase, subunit 5
NDUFA6: NADH dehydrogenase (Ubiquinone) 1 alpha subcomplex 6
NDUFA9: NADH dehydrogenase (Ubiquinone) 1 alpha subcomplex 9
NDUFB8: NADH dehydrogenase (Ubiquinone) 1 beta subcomplex 8
OxPhos: oxidative phosphorylation system
qRT–PCR: quantitative real-time PCR
ROS: reactive oxygen species
SA-ß-gal: senescence-associated beta-galactosidase
SDHA: succinate dehydrogenase complex subunit A
UCP1: uncoupling protein 1
UCP2: uncoupling protein 2
UQCRC1: ubiquinol-cytochrome-c reductase complex core protein 1
UQCRC2: ubiquinol-cytochrome-c reductase complex core protein 2

## Supporting Information

Figure S1 Upper panel: Human diploid fibroblasts were infected by lentiviruses carrying non-targeting (scr) or Beclin1-targeting (bec-1KD) shRNAs and grown in DMEM containing either 30 mg L^−1^ (30 MET) or 1 mg L^−1^ (1 MET) of methionine, as indicated.
