# Supplementary material for: Methionine restriction slows down senescence in human diploid fibroblasts
Source: Aging Cell. 2014 Oct 1;13(6):1038–48. doi: 10.1111/acel.12266 (PMC4326930; doi:10.1111/acel.12266)

**Supplementary Data**

**to**

**Methionine restriction slows down senescence in human diploid fibroblasts**

Rafał Kozieł^1^, Christoph Ruckenstuhl^2^, Eva Albertini, Michael Neuhaus^1^, Christine Netzberger^2^, Maria Bust^3^, Frank Madeo^2^, Rudolf J. Wiesner^3,4,5^, and Pidder Jansen-Dürr^1^

**Supplementary Methods**

Lentiviral vectors carrying two different shRNAs directed against Beclin1 were prepared as described and used to infect Human diploid fibroblasts (HDF) at a MOI of 2. Cells were grown in DMEM containing either 30 mg/l (30 MET) or 1 mg/l (1 MET) of methionine. The number of cumulative population doublings (cPDL) was calculated at regular intervals, as described in Material and methods. Beclin1 expression was determined by immunoblot using Beclin1 antibodies (Sigma, St.Louis, USA).

**Supplementary Figure 1**

*Upper panel:* Human diploid fibroblasts were infected by lentiviruses carrying non-targeting (scr) or Beclin1-targeting (bec-1KD) shRNAs and grown in DMEM containing either 30 mg/l (30 MET) or 1 mg/l (1 MET) of methionine, as indicated. After 60 days in culture, lysates were prepared and probed with antibodies to Beclin1; alpha-tubulin served as loading control. Shown are results from two independent experiments.

*Lower panel:* Human diploid fibroblasts were infected by lentiviruses carrying non-targeting (scr) or Beclin1-targeting (bec-1KD) shRNAs and grown in DMEM containing either 30 mg/l (30 MET) or 1 mg/l (1 MET) of methionine, as indicated. The number of cumulative population doublings (cPDL) was calculated at regular intervals. Growth curves representing three independent experiments are shown.

**Supplementary Reference**

Mück, C., Micutkova, L., Zwerschke, W., and Jansen-Dürr, P. (2008) Role of insulin-like growth factor binding protein-3 in human umbilical vein endothelial cell senescence. Rejuvenation Res. *11*, 449-453

**Figure S1**


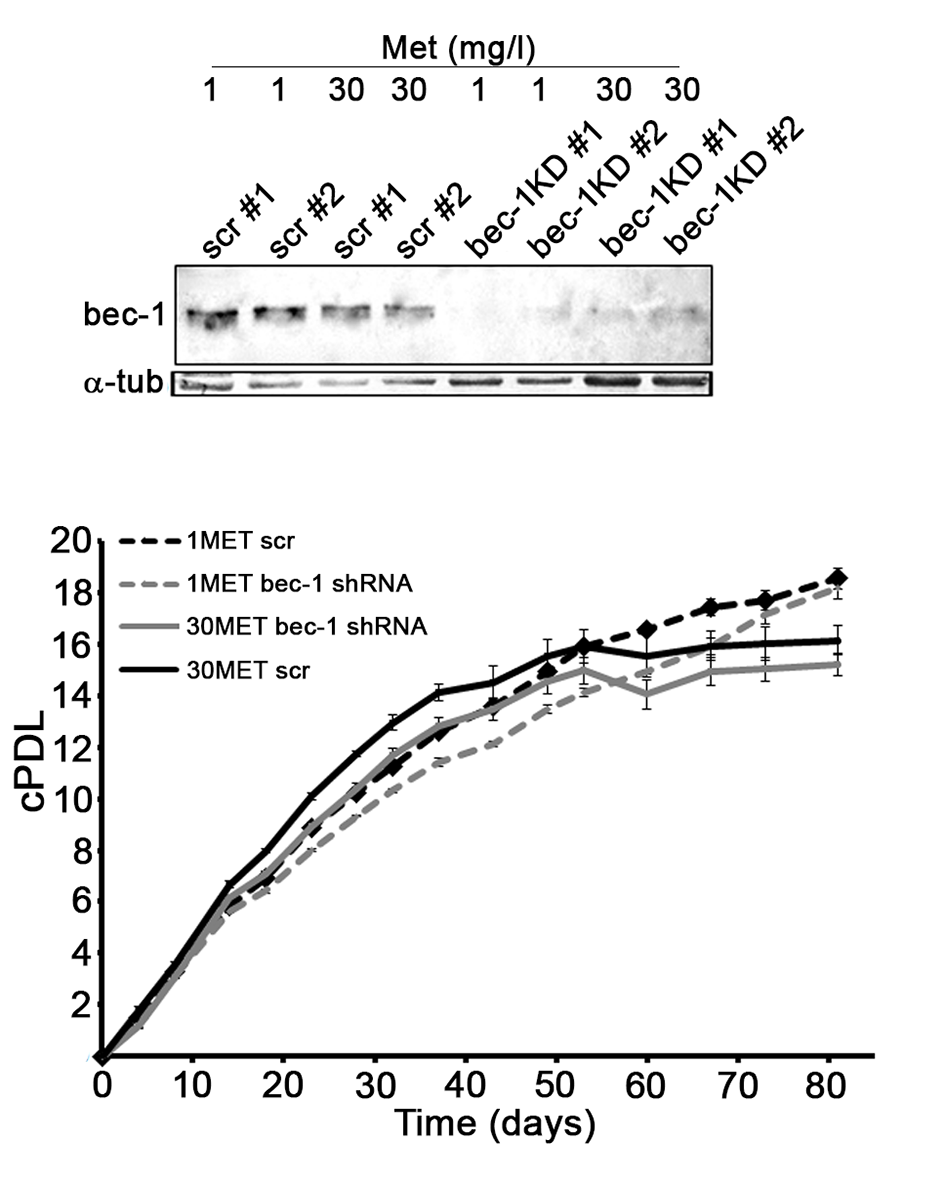

Supplement: Figure S1 — Upper panel: Human diploid fibroblasts were infected by lentiviruses carrying non-targeting (scr) or Beclin1-targeting (bec-1KD) shRNAs and grown in DMEM containing either 30 mg L−1 (30 MET) or 1 mg L−1 (1 MET) of methionine, as indicated. [file acel0013-1038-sd1.doc]
